# Comparative evaluation of prevention of demineralization of artificial enamel caries treated with two fluoride varnishes and 38% SDF in primary teeth: an in vitro study

**DOI:** 10.1186/s12903-023-02799-1

**Published:** 2023-02-17

**Authors:** Alshaimaa Mashhour, Gehan Allam, Mariem Wassel

**Affiliations:** Pediatric Dentistry and Dental Public Health Department, Faculty of Dentistry, Ain Shams University, Organization of African Unity St.-Abbasia, Cairo, 11566 Egypt

**Keywords:** Fluoride varnish, SDF, CPP-ACP, TCP, EDX, Polarized light microscope, Primary teeth

## Abstract

**Background:**

This study aimed to compare the effect of Clinpro™ White varnish containing 5% sodium fluoride (NaF) and functionalized tricalcium phosphate, MI varnish containing 5% NaF and casein phosphopeptide-amorphous calcium phosphate (CPP-ACP), and 38% Silver diamine fluoride (SDF) in preventing demineralization of treated white spot lesions (WSLs) in enamel of primary teeth.

**Methods:**

Forty-eight primary molars with artificial WSLs were allocated into four groups as follows: Group 1: Clinpro white varnish, Group 2: MI varnish, Group 3: SDF, and Group 4: control (no treatment). The three surface treatments were applied for 24 h and then enamel specimens were subjected to pH cycling. Thereafter, the mineral content of specimens was evaluated by Energy Dispersive X-ray Spectrometer and the lesion depth was assessed via Polarized Light Microscope. One-way ANOVA followed by Tukey’s post hoc test were used at *p* ≤ 0.05 to identify significant differences.

**Results:**

Insignificant difference in mineral content was observed among treatment groups. Treatment groups exhibited significantly higher mineral content compared to control except for Fluoride (F). MI varnish showed the highest mean calcium (Ca) ion content (66.57 ± 0.63), and Ca/P (2.19 ± 0.11), followed by Clinpro white varnish, and SDF. MI varnish also displayed the highest phosphate (P) ion content (31.46 ± 0.56), followed by SDF (30.93 ± 1.02), and Clinpro white varnish (30.53 ± 2.19). Fluoride content was highest in SDF (0.93 ± 1.18), followed by MI (0.89 ± 0.34) and Clinpro (0.66 ± 0.68) varnishes. Significant difference in lesion depth was observed among all groups (*p* < 0.001). The lowest mean lesion depth (µm) was found in MI varnish (226.23 ± 44.25) which was significantly lower than Clinpro white varnish (285.43 ± 44.70), SDF (293.32 ± 46.82), and control (576.69 ± 42.66). Insignificant difference in lesion depth was found between SDF and Clinpro varnish.

**Conclusions:**

In primary teeth, WSLs treated with MI varnish displayed better resistance to demineralization compared to WSLs treated with Clinpro white varnish and SDF.

## Background

White spot lesion (WSL) is considered the first sign of dental caries. It clinically appears as a chalky white opacity and is considered the earliest macroscopic evidence of enamel caries. WSLs are characterized by a porous subsurface enamel while the outermost enamel layer covering the lesion remains relatively intact and appears radiopaque. Control of WSLs includes prevention of demineralization and enhancement of remineralization to arrest lesion activity and improve esthetics [1].

Fluoride varnish is one important WSLs’ management strategy, especially in children, due to their safety and ease of application. Fluoride varnishes promote the growth of calcium fluoride on tooth surfaces which acts as an intraoral fluoride reservoir releasing calcium and fluoride ions when the oral pH drops [2]. To increase the effectiveness of fluoride varnishes, some manufacturers have added calcium (Ca) and inorganic phosphate (P) ions to enhance the precipitation of fluoro-hydroxyapatite by increasing its constituting ions bioavailability [3].

Clinpro white varnish (3 M Espe, USA) combines 5% NaF with a unique functionalized tricalcium phosphate (fTCP). In Clinpro white varnish, TCP is combined with fumaric acid producing free phosphate and fumaric acid-protected calcium oxide. The calcium protected with fumaric acid does not react with fluoride (F) till varnish application. When the varnish is applied on the teeth surfaces, it slowly dissolves releasing Ca, P and F ions for up to 24 h [4].

MI varnish (GC, Tockyo, Japan) is a one fluoride varnish product that contains 5% NaF and 2% RECALDENT™ [casein phosphopeptide-amorphous calcium phosphate (CPP-ACP) nanocomplexes]. CPP-ACP is an amorphous form of calcium phosphate (ACP) stabilized by a casein phosphopeptide (CPP) derived from milk casein. The varnish sets when it contacts saliva and slowly releases F and CPP-ACP. This increases the bioavailability of F, Ca and P ions enhancing remineralization [5]. According to the manufacturer, the size of CPP-ACFP nanocomplexes are less than 2 nm enhancing penetration into biofilm, enamel, and dentinal tubules [6].

The use of silver diamine fluoride (SDF) has increased in recent years as a minimally invasive approach for caries management. Its high F content (44,800 ppm) enhances remineralization, while its silver content kills cariogenic bacteria and thus lesion arrest is promoted [7]. When applied on carious enamel lesions, SDF interacts with hydroxyapatite in the tooth structure to generate calcium fluoride and silver phosphate which improves the mineral density of enamel, increases the hardness of hydroxyapatite crystals, and reduces the depth of the carious lesion [7, 8]. Moreover, further mineral loss from demineralized tooth structures was found to decrease following SDF application, possibly due to incorporation of silver nanoparticles into treated hydroxyapatite [9]. Like fluoride varnish, SDF’s ease of use and effectiveness have made SDF application a popular treatment in pediatric dentistry.

Despite the availability of different remineralizing agents, there is inconclusive evidence about the superiority of a specific agent specifically in primary teeth. Moreover, studies reporting on the effectiveness of SDF in comparison to fluoride varnishes containing different sources of calcium phosphate are scarce. Only one in-vitro study investigated those products, however on bovine dentin [4].

Therefore, the aim of the present study was to compare the mineral content and lesion depth of artificial enamel caries in primary teeth treated with Clinpro white varnish, MI varnish and 38% SDF after being subjected to pH cycling. The null hypothesis was that there would be no difference in the remineralization potential among treatment groups.

## Methods

### Sample size estimation

Based on the null hypothesis and by adopting an alpha level of 5%, a beta level of 20% i.e. power = 80% as well as an effect size of 0.60 based on the results of a previous study [10]; the predicted sample size (n) was 48.

### Enamel specimens’ preparation

Forty-eight sound primary molars, that were freshly extracted for orthodontic reasons or naturally exfoliating, were included. Teeth were cleaned, polished with a non-fluoride containing polishing paste and examined under a stereomicroscope to exclude teeth with developmental defects, cracks, or enamel demineralization in the buccal surface. Teeth were stored in distilled water until use [11, 12]. A 4 × 4 mm^2^ square adhesive tape was placed on the center of the buccal surface of each tooth. The entire tooth was then coated with an acid resistant nail varnish (Amanda A.R.E). A 4 × 4 mm^2^ enamel window was created by removing the adhesive paper [12].

Subsurface enamel lesions were created by submerging each tooth in 10 ml of a demineralizing solution (pH 4.4) for 4 days inside a light resistant container [11] until a distinct visual change in enamel in both dry and wet conditions was evident (ICDAS score 2) [13].

### Treatment protocols and pH cycling

After induction of WSLs, each tooth was rinsed with 10 ml deionized water and dried with a stream of compressed air. Teeth were randomly divided into four groups by a study independent operator as follows:

- *Group 1* In which a thin coating of Clinpro white varnish was applied to exposed enamel using a micro-brush.
- *Group 2* In which a thin coating of MI varnish was applied to exposed enamel using a micro-brush.
  In groups 1 and 2, teeth were allowed to air dry for 5 min before being placed in artificial saliva for 24 h [12]. Varnishes were carefully removed from all specimens using a cotton swab dipped in acetone to avoid harming the enamel surface. Teeth were then rinsed with deionized water for 1 min before being subjected to pH cycling [11].
- *Group 3* In which 38% SDF was applied with a micro-brush to exposed enamel for two minutes. Teeth were washed with 10 ml deionized water for 30 s, dried with compressed air and stored in artificial saliva for 24 h. Thereafter, teeth were rinsed with deionized water for 1 min before pH cycling [12].
- *Group 4 (Control group)* After creating WSLs, teeth were left untreated and were stored in artificial saliva for 24 h. Teeth were rinsed with deionized water for 1 min before pH cycling.

### pH cycling

All specimens were subjected to 9 days of pH cycling (8 days de/remineralization + 1-day remineralization). Demineralization/Remineralization cycles consisted of teeth immersion in 60 ml of demineralizing solution for 4 h followed by immersion in 60 ml of remineralizing solution for 20 h. This cycle was repeated for 8 days consecutively, and new solutions were used daily. After each cycle, specimens were washed with deionized water before progressing to the next cycle. On day 9, the specimens were immersed in the remineralization solution for 24 h [11, 14]. Details of materials used in the study are listed in Table 1.

**Table 1:** Materials used in the study

Material	Composition
Clinpro™ White varnish (3 M Espe, MN, USA)	30–75% pentaerythritol glycerol ester of colophony resin, 10–15% n-hexane, 1–15% ethyl alcohol, 5% sodium fluoride, 1–5% flavor enhancer1–5% thickener, 1–5% food grade flavor, < 5% modified tricalcium phosphate
MI varnish (GC, Tokyo, Japan)	30–50% polyvinyl acetate, 10–30% hydrogenated rosin, 20–30% ethanol, 5% sodium fluoride, 1–5% CPP-ACP, 1–5% silicon dioxide
e-SDF (Globus Medisys Gujarat, India)	25% silver, 8% ammonia, 5% fluoride, and 62% water. This is referred to as 38% SDF
Demineralization solution	(2.20 mmol/L calcium chloride, 2.20 mmol/Lmonosodium_ phosphate, 1 mol/L potassium hydroxide and 0.05 mol/L acetic acid; pH 4.4)
Remineralization solutions	(1.5 mmol/L calcium chloride, 0.9 mmol/L monosodium- phosphate, 150 mmol/L potassium chloride; pH 7.0)
Artificial saliva	3.9 mM Na3 PO4, 4.29 mM NaCl, 17.98 mM KCl, 1.1 mM CaCl2 0.08 mM MgCl2,0.5 mM H2 SO4, 3.27 mM NaHCO3, and distilled water

### Mineral content measurement

After pH cycling, teeth were washed with deionized water and prepared for quantitative elemental analysis using energy dispersive X-ray spectrometer (EDX) (Inspect S Manufacturer, FEI Company, Netherlands) with an accelerating voltage of 30 kV, magnification up to 10^6^ and a 1 nm gun’s resolution. Specimens were fixed with a double adhesive tape to a sample holder so that the enamel window was facing upward. Three different readings were obtained from the center of each enamel window and an average was calculated for each specimen. The weight percent of calcium (Ca), fluoride (F) and phosphorus (P) in enamel surface was assessed as well as the Ca/P ratio [12, 15].

### Lesion depth measurement

After EDX assessment, teeth were qualitatively assessed for lesion depth with a polarized light microscope (PLM) (PRIOR scientific, PriorLux POLTM) at a magnification of × 100. Using a slow-speed diamond saw and ample water spray, teeth were longitudinally sliced into mesial and distal halves from the center of the 4 × 4 mm^2^ window. For each tooth, one tooth half was selected using a toss of a coin. Tooth halves were then reduced to a thickness of 100–150 µm using wet 150 grit silicon carbide papers [11, 14]. Image J analysis software (Java-based image processing program) was used to measure the depth of demineralized zones. In each specimen, the deepest area of enamel demineralization was selected, and the lesion depth was measured in (µm) via drawing 3 perpendicular lines to the outer enamel surface at three distinct places (at the center of the deepest area of the lesion and at the peripheries of this area). The mean was determined and recorded as the specimen's lesion depth [11, 14].

### Statistical analysis

Data were analyzed using one-way ANOVA followed by Tukey’s post hoc test. The significance level was set at *p* ≤ 0.05 within all tests. Statistical analysis was performed with R statistical analysis software version 4.1.2 for Windows.^1^

## Results

### I-EDX analysis

Mean and standard deviation (SD) values of weight percentage (%) for different elements are presented in Table 2. Mineral content analysis showed the following:

- *Ca weight %* The highest value was found in MI varnish (66.57 ± 0.63), followed by Clinpro white varnish (66.23 ± 0.51), then SDF (64.51 ± 0.52), while the lowest value was found in the control group (34.68 ± 3.29). Insignificant difference was found among the treatment groups, yet the control group differed significantly with all other groups (*p* < 0.001).
- *P weight %* The highest value was found in MI varnish (31.46 ± 0.56), followed by SDF (30.93 ± 1.02), then Clinpro white varnish (30.53 ± 2.19), while the lowest value was found in the control group (18.33 ± 1.90). Post hoc pairwise comparisons showed the control group to have a significantly lower value than other groups (*p* < 0.001), while insignificant difference was found among the treatment groups.
- *Ca/P* The highest value was found in MI varnish (2.19 ± 0.18), followed by Clinpro white varnish (2.11 ± 0.05), then SDF (2.09 ± 0.06), while the lowest value was found in the control group (1.90 ± 0.18). Post hoc pairwise comparisons showed the control group to have a significantly lower value than other groups (*p* = 0.003), while insignificant difference was found among the treatment groups.
- *F weight %* The highest value was found in SDF (0.93 ± 1.18), followed by MI varnish (0.89 ± 0.34), then Clinpro white varnish (0.66 ± 0.68), while the lowest value was found in the control group (0.54 ± 0.52). Non-significant difference was found among all groups.

**Table 2:** EDX analysis

Element	Clinpro white	SDF	MI varnish	Control	*p*-value
Ca	66.23 ± 0.51^A^	64.51 ± 0.52^A^	66.57 ± 0.63^A^	34.68 ± 3.29^B^	< 0.001*
P	30.53 ± 2.19^A^	30.93 ± 1.02^A^	31.46 ± 0.56^A^	18.33 ± 1.90^B^	< 0.001*
Ca/P	2.11 ± 0.05^A^	2.09 ± 0.12^A^	2.19 ± 0.11^A^	1.90 ± 0.18^B^	0.003*
F	0.66 ± 0.68^A^	0.93 ± 1.18^A^	0.89 ± 0.34^A^	0.54 ± 0.52^A^	0.703

Means with different superscript letters within the same horizontal rows are significantly different *; significant (*p* ≤ 0.05) ns; non-significant (p > 0.05)

### II- lesion depth

**Fig. 1:**
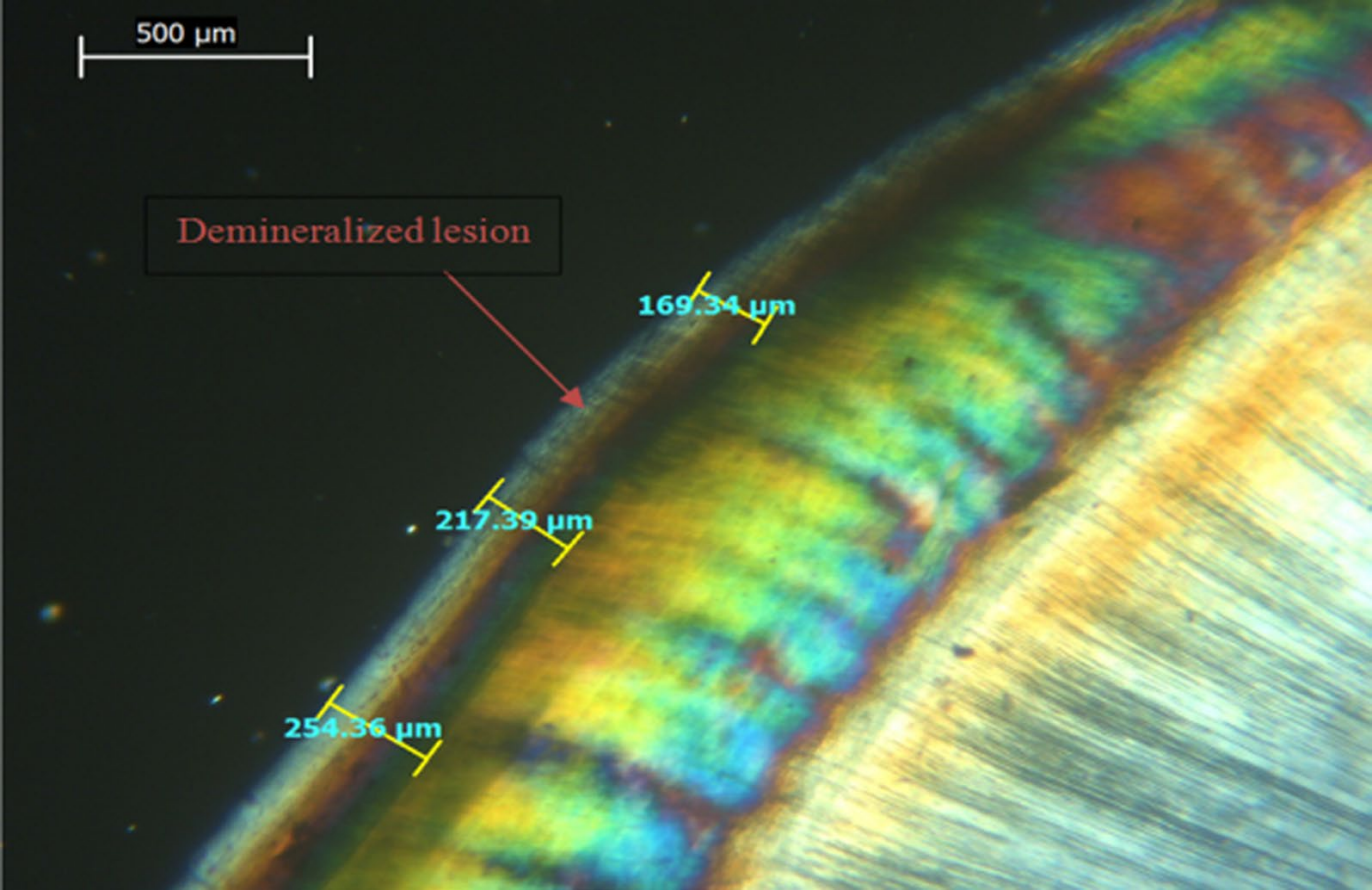
PLM image of a representative lesion from group 1 (clinpro™ white) showing lesion depth at 3 different areas

**Fig. 2:**
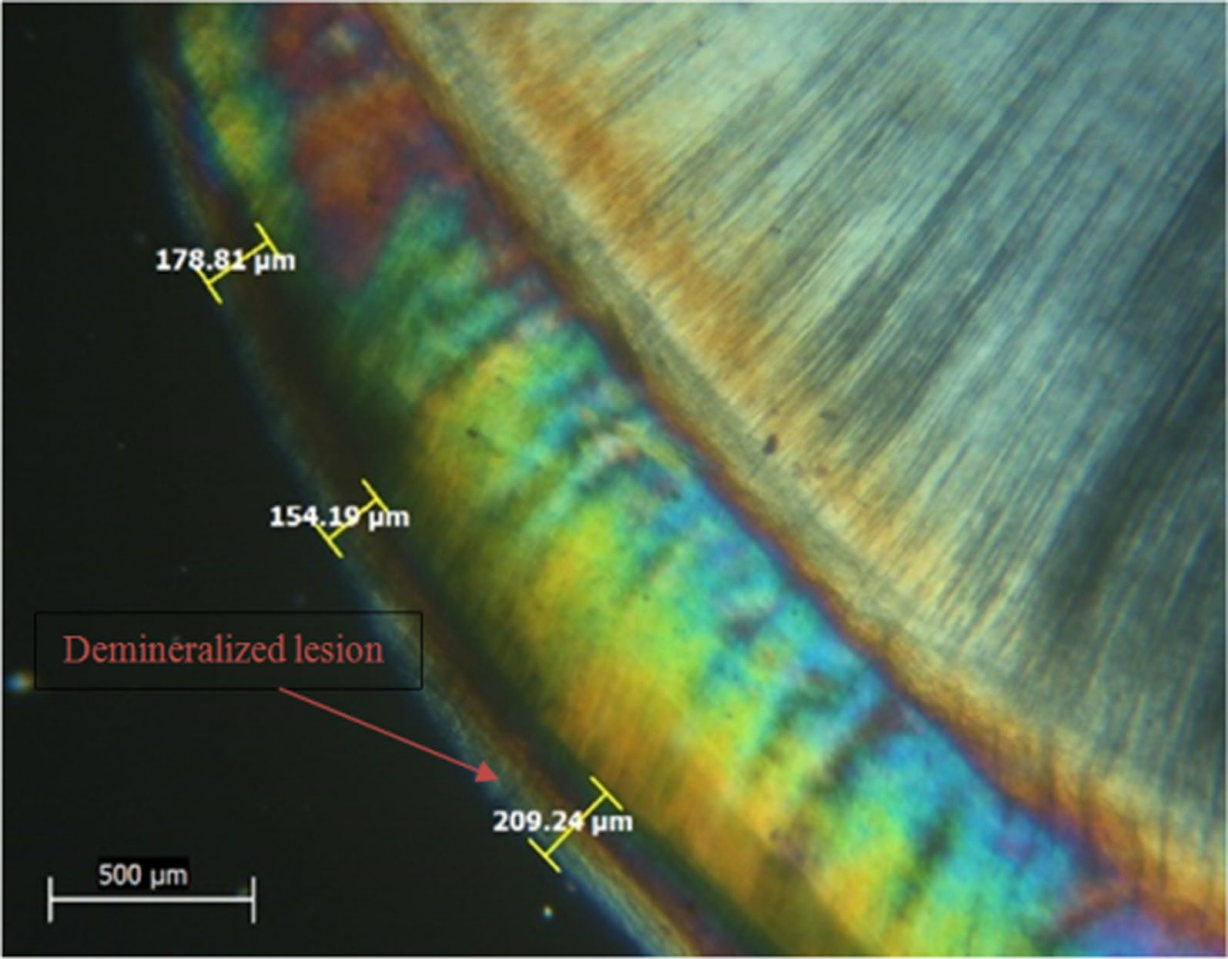
PLM image of a representative lesion from group 2 (MI varnish) showing lesion depth at 3 different areas

**Fig. 3:**
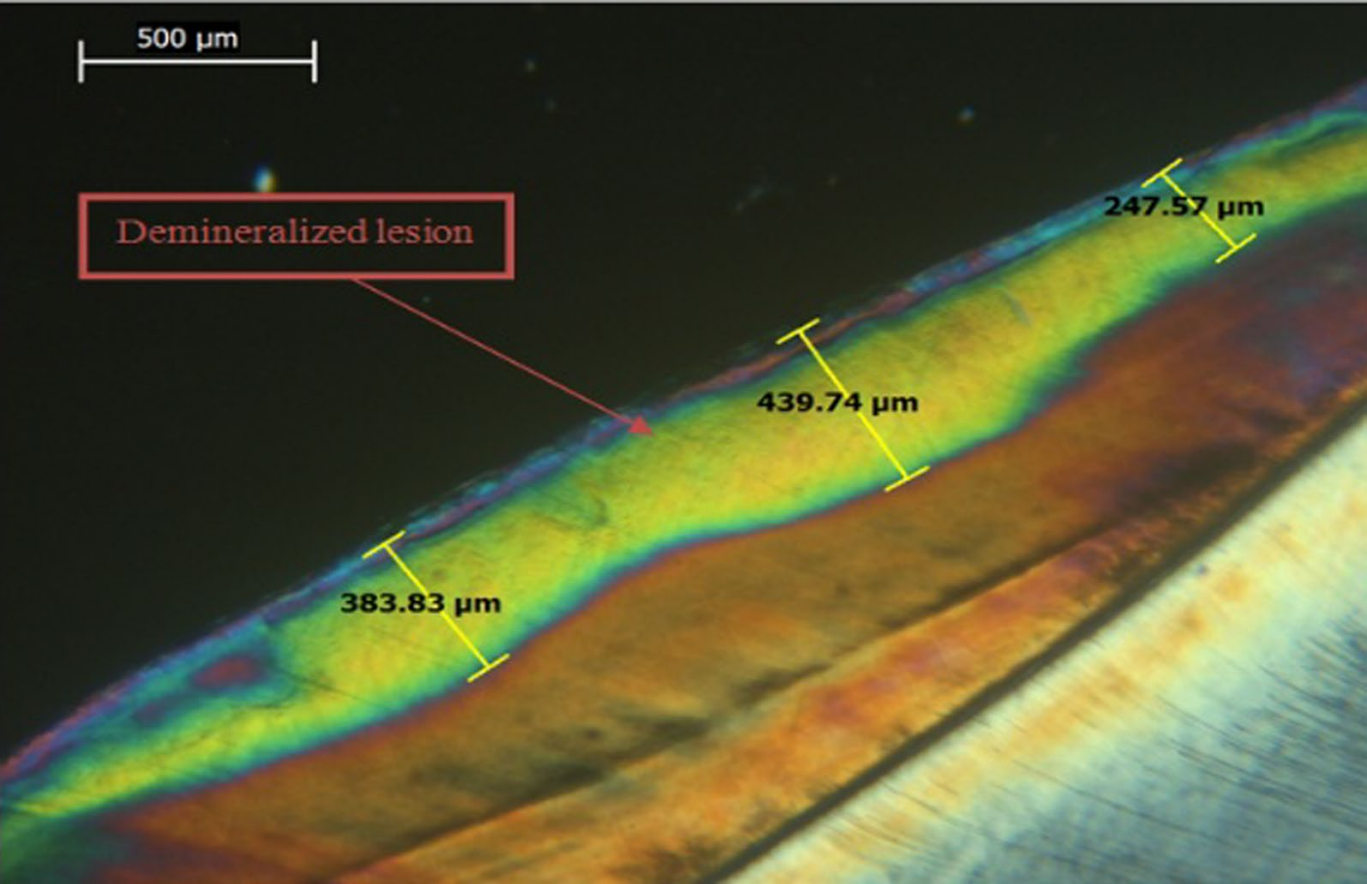
PLM image of a representative lesion from group 3 (SDF) showing lesion depth at 3 different areas

**Fig. 4:**
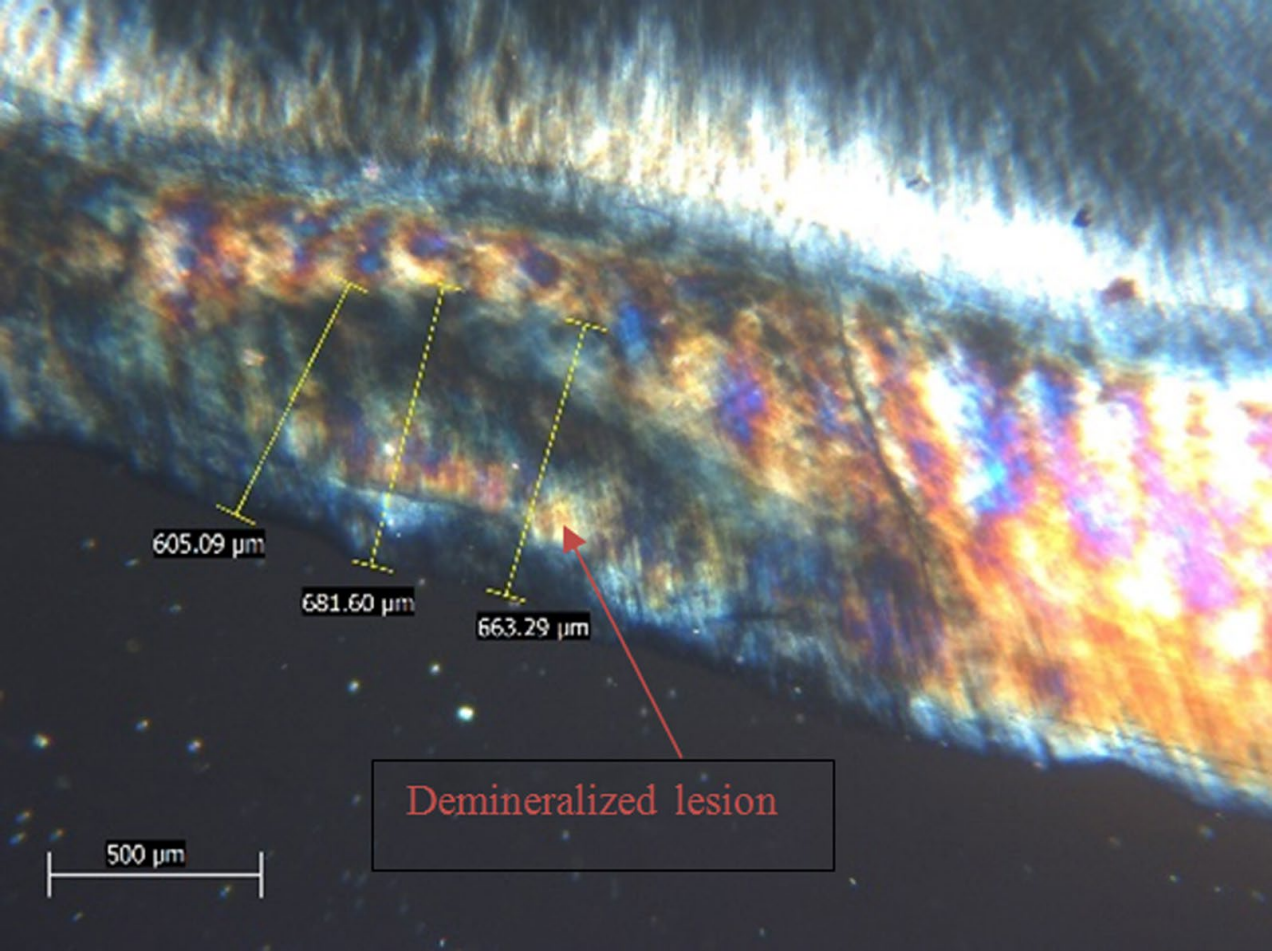
PLM image of a representative lesion from group 4 (control) showing lesion depth at 3 different areas

Mean and SD values of lesion depth (µm) for different groups are presented in Table 3. Representative PLM images for all groups are presented in Figs. 1, 2, 3 and 4. A significant difference was evident among groups (*p* < 0.001). The highest value for lesion depth was found in the control group (576.69 ± 42.66), followed by SDF (293.32 ± 46.82), then Clinpro white varnish (285.43 ± 44.70), while the lowest value was found in MI varnish (226.23 ± 44.25). SDF and Clinpro white varnish had significantly higher lesion depth values than MI varnish (*p* < 0.001).

**Table 3:** lesion depth (µm) for different groups

Lesion depth (µm) (mean ± SD)				*p*-value
Clinpro white	SDF	MI	Control	
285.43 ± 44.70^B^	293.32 ± 46.82^B^	226.23 ± 44.25^C^	576.69 ± 42.66^A^	< 0.001*

Means with different superscript letters within the same horizontal rows are significantly different *; significant (*p* ≤ 0.05) ns; non-significant (*p* > 0.05)

## Discussion

In early enamel lesions, both fluoride varnish and SDF have been suggested as treatment strategies, especially in young children, due to their ease of application and safety [3, 12, 16, 17]. The effect of SDF compared to NaF varnish on enamel remineralization was previously investigated [12, 18]. Yet, up to our knowledge, our study is the first to compare the effect of SDF and fluoride varnishes containing calcium-phosphate on WSLs in primary teeth.

Our results demonstrated that the three investigated topical treatments were able to remineralize demineralized enamel of primary teeth. MI varnish followed by Clinpro white varnish were better than SDF in terms of Ca and P content, Ca/P ratio, as well as lesion depth. Although EDX analysis revealed insignificant differences in Ca, P, and F ions content as well as Ca/P among all treatment groups, yet Ca content and Ca/P were higher in MI varnish followed by Clinpro white varnish. On the other hand, the control group showed significantly lower values for all variables except for F ion content. This indicates that the three topical agents exerted considerable remineralization of the WSLs, thus increasing their resistance to further demineralization when exposed to a subsequent acidic challenge. Thus, the null hypothesis is not rejected. The F content in the 3 agents could have contributed to the remineralization process by enhancing precipitation of minerals into the decalcified lesions [19]. Although F content in SDF is much higher than MI and Clinpro white varnishes, yet the adhesiveness of varnishes that allows prolonged contact with enamel as well as the availability and sustained release of F and other minerals may have contributed to the higher Ca and P content as well as Ca/P in MI varnish and Clinpro white groups. In agreement with the present findings, the effectiveness of MI varnish compared to other fluoride and calcium phosphate containing varnishes was reported in many studies [11, 20–24]. One study disclosed that MI varnish had a significantly higher capacity to remineralize decalcified enamel in terms of microhardness and mineral content compared to Clinpro white varnish [23]. Shen, et al. [24] reported that sound primary teeth enamel previously treated with MI varnish was more resistant to acid demineralization than specimens treated with Clinpro white varnish or a 5% NaF varnish where the % of inhibition of demineralization was significantly highest in MI varnish while lesion depth was significantly lowest compared to other groups. Shen, et al. [24] also reported that MI varnish exhibited the highest Ca, P, and F ions release compared to Clinpro white varnish as well as other calcium phosphate and fluoride containing varnishes.

Abufarwa, et al. [14] disclosed that MI varnish application could prevent enamel demineralization for up to 4 weeks. Cochrane et al. [25], also found that MI varnish significantly released higher F, Ca and P compared to Clinpro white varnish. The authors suggested that the low ion release of Clinpro white may be due to the low concentration of added fTCP or the low solubility of TCP.

CPP in MI varnish may have also contributed to a better remineralization by stabilizing CPP-ACFP nanoclusters preventing them from growing to the threshold size for phase transitions, thus keeping Ca and P ions freely accessible to diffuse into mineral-deficient lesions [26, 27]. Furthermore, casein contains amino acids that may have acted as acid buffers, thus provided treated specimens a better resistance to demineralization [21]. Another positive characteristic of MI varnish is that the CPP-ACFP nanocomplexes are electroneutral ion clusters thus they can rapidly diffuse out of the varnish and into subsurface lesions through intra-prismatic spaces [28].

Opposite to our findings, one study reported that a 5% NaF containing varnish and Clinpro white varnish achieved significantly better remineralization of demineralized enamel compared to MI varnish. However, in this study, varnishes were applied for only 6 h before treated specimens were subjected to pH cycling [29].

In the present study, Clinpro white varnish exhibited more Ca and Ca/P ratio compared to SDF which may be related to the TCP content of Clinpro white as well as its manufacturing process where fTCP is produced by functionalizing TCP with silica which promotes linking with hard tissue defects in acidic conditions [29, 30]. Other studies also confirmed that addition of TCP to fluoridated products achieved better remineralization than products containing fluoride only [11, 24, 31].

Nevertheless, a systematic review concluded that the potential of fluoride varnishes to arrest dental caries is not satisfactory, and that topical products with higher fluoride concentrations may be more effective [32]. The potential of SDF to remineralize and arrest enamel and dentin caries is well documented where a Ca and P rich layer was found on the surface of caries arrested lesions [9, 16]. Although most of the evidence pertaining to SDF comes from studies concerned with arresting dentin caries, its effectiveness in remineralizing initial enamel lesions was also reported [17]. Eventually, a systematic review and meta-analysis indicated that SDF application to decayed primary teeth can effectively prevent dental caries in the entire dentition compared to fluoride varnish [33].

Our findings showed SDF-treated specimens to have the highest F content. This finding might be attributable to the flowability of the SDF solution which makes complete contact with the enamel surface and thus delivers a high amount of F to demineralized enamel in a short period of time. It could also be due to the SDF’s high F content which is nearly double that of Clinpro white and MI varnishes [12]. It has been demonstrated that SDF can penetrate decalcified enamel, thereby, it acts as a fluoride reservoir enhancing remineralization and preventing further demineralization [34].

The cariostatic effect of SDF is not yet fully understood. It is suggested to be a result of antibacterial and remineralizing effects. Given that no bacterial biofilm was used in the present study, the noted SDF effect is therefore related to its remineralizing effect. It is suggested that an insoluble layer is formed after SDF application which consists of calcium fluoride, silver phosphate, silver chloride, and fluorapatite. This layer protects against further demineralization and prevents lesion progression. Yet this layer can also hinder minerals deposition inside the body of the lesion [12, 17]. This may explain the higher lesion depth of SDF group in the present study compared to MI and Clinpro white varnishes.

Opposite to our results, Yu et al. [12] demonstrated that lesion depth was lower in SDF treated subsurface enamel lesions compared to a 5% NaF varnish. However, in the latter study, fluoride varnish was applied for only 60 min. Additionally, the bioavailability of Ca and P in varnishes used in the current study may account for this inconsistency. Notably, Yu et al. [12] also pointed out that insoluble silver chloride was formed on the lesion surface, and this may be responsible for the increased hardness of WSLs. The authors also reported that silver nanoparticles were incorporated into the hydroxyapatite crystal in SDF-treated teeth which in turn can suppress bacterial adhesion thus preventing lesion progression [12].

Thus, although results of our study indicated that SDF showed lower Ca and Ca/P as well as deeper lesions than the tested varnishes, its clinical application to WSLs may increase resistance to bacterial induced demineralization more than the investigated varnishes. Thus, SDF may be of benefit in non-compliant patients. This also highlights the need to investigate SDF and fluoride varnishes in the presence of bacteria to mimic the oral environment.

Previously, Romão, et al. [17] suggested that SDF can be used for communities with limited financial resources, high caries risk individuals or those with poor compliance because SDF can immediately release all its fluoride content thus achieving more rapid arrest compared to fluoride varnishes.

A limitation of the present study is that in-vitro remineralization may be quite different when compared to the dynamic complex biological systems that occur in the oral cavity. Additionally, the antibacterial effect of the three tested agents was not taken into consideration where the clinical remineralization of carious lesions may be enhanced through availability of free mineral ions as well as the antimicrobial action of the used agents. Therefore, due to the evident limitations of in-vitro investigations, caution must be taken when making direct interpretations to clinical circumstances. Another limitation was that lesion depth of the treatment groups was not measured directly after demineralization and later compared to the post-treatment and pH cycling scores. Instead, demineralization was confirmed visually using the ICDAS-II relying on the significantly strong positive correlation that ICDAS has with histological lesion depth, where score 2 indicates a demineralized enamel that extends beyond half the enamel thickness [35]. Thereafter, post-treatment and pH cycling scores were compared to a control group that received no treatment as in some previous studies [11, 14, 17].

## Conclusion

Within the limitation of the present study, the investigated remineralizing agents were all found to have a positive impact on prevention of enamel demineralization of treated WSLs in primary teeth. Fluoride varnish containing CPP-ACP (MI varnish) was the most effective in enhancing acid resistance. Yet, clinical trials are still required to verify the data obtained from this in-vitro investigation.

## Data Availability

The datasets generated and/or analyzed during the current study are available from the first author upon request.

## Footnotes

1 R Core Team (2022). R: A language and environment for statistical computing. R Foundation for Statistical Computing, Vienna, Austria. URL https://www.R-project.org/.
